# Smoking & autonomy: the generational tobacco endgame

**DOI:** 10.1007/s40592-024-00207-0

**Published:** 2024-10-03

**Authors:** Shazeea Mohamed Ali

**Affiliations:** 1 Monash University, Clayton, VIC 3168 Australia; 2 Department of Geriatric Medicine, Austin Health, Heidelberg, VIC 3084 Australia

**Keywords:** Smoking, Tobacco, Cigarette, Nicotine, Autonomy, Paternalism, Public health ethics

## Abstract

New Zealand and Malaysia have abandoned plans to introduce a generational smoking ban because of concerns that such a policy is incompatible with liberal democracy as it undermines autonomy. This paper challenges this claim by showing that smoking is not an autonomous act. Autonomy requires a deliberation of preferences, wills and inclinations. This does not occur in smokers because of three related factors: nicotine addiction, cognitive biases and psychosocial development in addiction. Nicotine addiction results in strong physical and psychological desires to seek pleasure and to avoid withdrawal. This is further potentiated by conditioned behaviour. Cognitive biases explain why smokers act in ways that are detrimental to their health. Psychosocial development explains how the brains of smokers are unable to make rational decisions. This combination renders smokers unable to reflect on their actions and thus act autonomously. This stance is compatible with Mill’s view that actions that devalue autonomy cannot be considered autonomous. Defenders of liberalism should not be quick to dismiss a smoking ban and can instead foster autonomy by supporting it.

## Introduction

A generational smoking ban is a policy that seeks to prohibit the use of tobacco products by people born after a specified year. It can take the form of a ban on the sale of tobacco products to the target population, or a ban on the possession and use of tobacco by the target population. This approach aims to achieve a tobacco endgame, an ambitious but controversial goal. In the past year, both New Zealand and Malaysia abandoned plans to implement smoking bans for future generations. New Zealand’s ban, set to take effect in July 2024, was repealed when a new coalition government came into power in late 2023. Malaysia faced obstacles in introducing the bill, including concerns raised by its attorney general, which ultimately led to its abandonment. The United Kingdom’s ability to pass a generational smoking ban remains to be seen.

The recently formed conservative administration in New Zealand comprises individuals who have consistently opposed the ban, contending that it is a matter of “freedom of choice” (Bajaj 2023). In Malaysia, the concerns expressed were that the ban would violate the principles of equality before the law and personal liberty enshrined in the country’s constitution (CodeBlue 2023). The opposition to the ban in both countries is rooted in the argument that it is incompatible with a democratic state. Both Malaysia and New Zealand are parliamentary democracies modeled after the Westminster system, and their constitutions guarantee the liberties of their citizens. Autonomy is a fundamental principle of a liberal state and has significant normative importance.

Proponents of generational smoking bans ground their support for a ban in utilitarianism or the harm principle. However, I propose a different approach by arguing that a smoking ban does not infringe upon individual autonomy because smoking is not an autonomous act. To support this thesis, I first define autonomy and identify the necessary conditions for an act to be considered autonomous. In the second section, I examine three related concepts that undermine autonomy in smokers: nicotine addiction, cognitive bias, and psychosocial development. I combine these three factors to argue that smoking is not an autonomous act. Finally, I address potential criticisms and conclude my argument.

## Autonomy

“Autonomy” is derived from the Greek *autos* meaning “self” and *nomos* meaning “rule, governance or law.” Its original use refers to the self-governance of independent Greek city-states. However, contemporary usage encompasses individuals (Beauchamp and Childress 2019). Individual autonomy is understood as a person’s ability to rule and decide for one’s self, without controlling external influences or impairment from factors such as diminished capacity or misinformation that could impede meaningful choice. (Beauchamp and Childress 2019; Christman 2020). Diminished autonomy means to be significantly controlled by others or incapable of deliberating and choosing based on one’s preferences (Beauchamp and Childress 2019). Young children have diminished autonomy both by virtue of their life decisions being made by their parents and by lacking the capacity to make some of those decisions.

Autonomy has taken on additional, more complex meanings, such as liberty rights, privacy, individual choice, freedom of the will, and living life according to one’s own preferences. However, it is widely accepted that two conditions are necessary for autonomy (Beauchamp and Childress 2019). They are:

1. Liberty: independence from controlling influences.
2. Agency: capacity for intentional action.

Different conceptions of autonomy differ with regard to how these conditions should be present and the extent to which they ensure the characterisation of an act as autonomous.

### Early work on autonomy: Kant and Mill

Modern notions of autonomy trace their roots to the Enlightenment, particularly to the works of Immanuel Kant and John Stuart Mill (Colburn 2022). Kant believed that rational beings have the capacity to make moral law. The Categorical Imperative compels a moral agent to act according to “that maxim through which you can at the same time will that it become a universal law” (Johnson and Cureton 2022). Thus, establishing this moral law is an act of pure autonomous will unconstrained by the agent’s preferences or social forces (Stoljar 2006). John Rawls further emphasized that rational moral agents ought to formulate moral principles from behind a “veil of ignorance” wherein they have no knowledge of their personal circumstances, such as their own ethnicity, gender, age or wealth. This allows them to establish fair and unbiased principles without favouring any particular group they may represent (Taylor 2022).

This notion of autonomy is an ideal concept. It sets an extremely high standard for autonomous acts, as many actions that are typically considered autonomous are rendered nonautonomous by this definition. Human beings exist in a physical and social environment, and moral agents routinely make decisions that consider their own preferences, the preferences of those around them, and the constraints of the physical word.

Nevertheless, acting authentically was recognised as essential to human excellence by later philosophers. Mill posited that the expression of autonomy must include the cultivation of individuality and an authentic self by a moral agent (Donner 2009). This did not preclude a moral agent’s character from being shaped by their culture but rather stipulated a process of critical reflection and scrutiny of options to prevent mere conformity to societal norms (Donner 2009). This philosophical foundation laid the groundwork for subsequent discussions to determine whether an individual’s proclaimed values or desires are genuinely their own.

### Procedural accounts of autonomy

The most widely recognised procedural account of autonomy is the structural theory developed by Frankfurt (1971). According to this theory, an agent is autonomous with respect to their first-order desires as long as they are endorsed by second-order desires (Christman 2020; Frankfurt 1971; Stoljar 2006). If a moral agent’s first-order desire is to smoke a cigarette, they are only considered autonomous if they also have a second-order desire to want to smoke the cigarette. However, if they are compelled to smoke because of addiction and not desire, then the act is not autonomous (“the unwilling addict”). Procedural accounts of autonomy are content-neutral. This means that it is immaterial whether smoking is morally good or bad, and it does not matter where the first-order desire originates (whether it is internal or external) (Stoljar 2006). Instead, what matters is that an agent reflects on their desires and considers whether they wish to endorse or override them (Christman 2020).

Procedural accounts of autonomy are practical ways to understand autonomy as they illustrate the decision-making process. However, it is criticised for several reasons. First, procedural accounts result in an infinite regression. If a first-order desire is considered autonomous if it is endorsed by a second-order desire, it raises the question of where the second-order desire originates from, and whether there is a third-order desire *ad infinitum*. There is inadequate explanation as to why lower-order desires are considered autonomous for reasons that higher order desires are not. Second, procedural accounts fail to explain how self-reflection is not impacted by external factors and thus lack an explanation of how autonomy is protected from the influence of social conditions or the coercive forces of others (Taylor 2005).

### Substantive accounts of autonomy

The development of substantive accounts of autonomy was prompted by criticism of procedural accounts. Substantive accounts of autonomy argue that mere internal processes are insufficient for autonomous reasoning and that constraints on what can be considered autonomous are necessary. For example, without constraints, procedural accounts of autonomy may deem enslavement to be an autonomous choice if an individual’s first- and second-order desires align.

“Normative” substantive accounts link the failure of autonomy to the failure of competence or capacity in identifying and applying norms to one’s decision-making process (Stoljar 2006). These accounts of autonomy consider decisions made based on internalized false norms to be nonautonomous, as these beliefs have impeded one’s ability to distinguish right from wrong. They do not seek to explicitly spell out morality but focus on whether one’s upbringing or surroundings hinder the exercise of normative competence.

For example, if a moral agent has grown up in an environment where smoking is seen as normal behaviour, their desire to smoke may not be autonomous, not because smoking is morally wrong but because they have internalised a worldview in which everyone engages in it willingly. However, if a similar moral agent has grown up in an environment where smoking is associated with intensely negative experiences, such as illness and premature death leading to intergenerational trauma, and they then choose not to smoke because of this, this may also be nonautonomous. This is because, in both scenarios, the agents have blocked their capacity to criticise these false norms (false because there are harmful effects to smoking, but not all smoking is associated with significantly negative experiences).

The example above shows that the perception of an act can vary and raises the question of whether normative competency is underpinned by moral objectivity (Stoljar 2006). In terms of objective normative competency, contemporary Kantians reintroduce the “Kingdom of Ends” which compel moral agents to consider universal moral principles as a means to describe what these normative constraints should resemble (Reath 2006). They would not consider autonomous any act that appeared to devalue autonomy, such as relinquishing one’s autonomy to another.

Kant himself was a smoker (Kuehn 2014), but it is difficult to envision that if he were alive today, with knowledge of the harms of smoking, he would advocate for it to be a universal moral law. Most societies normatively discourage smoking, often by acknowledging its increased morbidity and mortality. Reducing smoking-related harms is a priority in most countries and for the World Health Organization.

### Relational accounts of autonomy

While substantive autonomy demands that a moral agent look inward to critique internalised beliefs, relational accounts of autonomy highlight the external factors that affect decision-making. Relational autonomy emerged from feminist critiques of traditional accounts of autonomy that, among other things, perceived the moral agent as self-sufficient, atomistic, and divorced from social relationships and care responsibilities. Existing theories of autonomy were not representative of the plurality of humanity, and were ignorant of psychoanalytical theories, and theories of power and agency (Mackenzie and Stoljar 2000).

At the core of relational autonomy is the argument that the individual is fundamentally a social being and that relationships of care and interdependence are valuable and morally significant. Since human nature is fundamentally social, any theory of autonomy must take this into account (Mackenzie and Stoljar 2000). The exercise of autonomy entails making decisions and acting in accordance with one’s values and preferences, while acknowledging and negotiating social relationships and the social context. As van der Eijk and Uusitalo (2016) explain, autonomy in addiction is “sociorelational” as discussed later.

Relational autonomy evaluates the existing social and political structures and their impact on development and expression autonomy. This involves identifying and addressing oppressive forces that interfere with autonomy (McLeod and Sherwin 2000), ranging in severity from peer pressure to gender oppression. When a moral agent is amidst peers who smoke, relational autonomy acknowledges the impact of this environment on their decision to smoke. The fact that the agent may be influenced by their surroundings does not necessarily make them nonautonomous, as they can still choose not to smoke. At a policy level, relational autonomy shifts the discussion from maximising an agent’s individual capacity for autonomous decision-making to modifying social conditions that eliminate social pressure in the first place (McLeod and Sherwin 2000).

Relational autonomy is an umbrella term of related theories that emphasize the significance of the individual as a socially embedded agent. Despite this shared belief, disagreement exists regarding the extent to which social context diminishes autonomy. This lack of clarity and consensus hampers the practical application of relational autonomy. Due to the theory’s focus on social determinants, relational autonomy risks absolving moral agents of agency and moral responsibility. Nevertheless, it is a helpful lens through which to examine issues in public health and healthcare, shedding light on areas that have previously been ignored.

### Summary

The relevance of Kantian ideal autonomy has diminished in today’s society due to its inability to account for the complex, multifaceted, social, and often conflicting nature of decision-making. It is crucial to establish a conception of autonomy that reflects daily decision-making and is recognizable by the public. John Christman proposes the concept of “basic autonomy,” which he defines as the “minimal status of being responsible, independent, and capable of speaking for oneself” (Christman 2020). For most moral agents, autonomy is procedural in nature, as self-governing agents express their will, which reflects their fundamental inclinations (Levy 2006). However, relational autonomy theorists question whether moral agents truly act “independently” and in accordance with their “fundamental” inclinations. Therefore, when evaluating autonomous choices, it is essential to consider the decision being made and the social context in which it is made.

## Nicotine, bias and psycosocial theories of addiction

### Nicotine

Harms from smoking occur because of the exposure to toxins in tobacco smoke. However, it is nicotine addiction that is the direct cause of disease (Benowitz 2010). Cigarette smoke inhalation leads to increased nicotine levels in the bloodstream via the lungs. Nicotine binds to nicotinic cholinergic receptors in the brain, ultimately leading to neurotransmitter release. When activated, nicotinic receptors release dopamine, glutamate, and γ-aminobutyric acid (GABA).

Dopamine is widely considered central to the development of addiction. It signals a pleasurable experience and thus reinforces behaviour that promotes the self-administration of the pleasurable agent. Dopamine is released in areas of the brain implicated in drug-induced reward (Dani and De Biasi 2001). Additionally, other compounds in tobacco and other neurotransmitters in the brain interact with dopamine, ultimately resulting in the increased excitation of dopaminergic neurons and enhanced responsiveness to nicotine (Benowitz 2010).

With chronic nicotine exposure, desensitisation occurs. This is the inactivation of the receptor caused by prolonged or repeated exposure (Ochoa et al. 1989). Research has shown that symptoms of cravings and withdrawal occur in smokers when desensitised receptors become responsive during periods of abstinence. Nicotine binding to these receptors during smoking alleviates these symptoms. To avoid these unpleasant symptoms, including stress and anxiety, most smokers maintain near-total saturation and desensitisation of nicotinic receptors (Benowitz 2010). As a result, the brain responds by increasing the number of binding sites on receptors, leading to tolerance. Consequently, the same amount of nicotine exposure leads to a diminished response, and greater doses are required to avoid symptoms of withdrawal.

Smoking is also highly modulated by conditioned behaviour. Smoking cessation is notoriously difficult, and the risk of relapse is maintained long after the withdrawal symptoms have subsided (Benowitz 2010). Smokers associate specific moods, behaviours, and situations, referred to as smoking-related cues, with the gratifying effects of nicotine.

Conditioning is a process in which a response, smoking, becomes more frequent as a result of reinforcement from smoking-related cues, with the reinforcement being a stimulus for the response (Murphy and Lupfer 2014). Smokers develop certain habits; for example, a cigarette with a cup of coffee, and when repeated frequently, they link the habit with the pleasurable effects of nicotine and establish smoking cues (Benowitz 2010). These cues are powerful urges to smoke and explain why the urge to smoke is sometimes overpowering. Physical aspects of smoking (heat, smell, taste) can also be smoking-cues, as can unpleasant moods as a result of withdrawal.

Nicotine use during pregnancy affects the developing foetal brain (Smith et al. 2010). Nicotine readily crosses the placenta and leads to changes in neuronal architecture by promoting cell death, altering nicotinic receptor expression, and affecting neurotransmitter system functions. Furthermore, maternal smoking increases the likelihood of a child engaging in smoking in the future as well as the risk of attention deficit hyperactivity disorder, anxiety, and depression (Smith et al. 2010).

Nicotine has a positive impact on mood, concentration, and task performance; however, this is attributed to its ability to alleviate withdrawal symptoms (Benowitz 2010). It induces pleasure owing to its effect on the release of dopamine and reduces stress and anxiety by preventing nicotine withdrawal. Addiction occurs as a consequence of positive reinforcement and avoidance of withdrawal symptoms and is heavily modulated by conditioned behaviour. At the biological level, prenatal exposure to nicotine can have long-term effects on the developing fetal brain, which may increase the likelihood of addiction and psychiatric disorders.

### Cognitive bias

It is difficult to claim that individuals who smoke are not generally aware of the harmful effects of smoking in the present age. However, despite this, individuals continue to smoke. Goodin (1989) contends that smokers are victims of a number of cognitive biases; optimism bias, anchoring fallacy, and time discounting. Optimism bias refers to the propensity to overestimate the likelihood of positive outcomes and to underestimate the likelihood of negative outcomes (Sharot 2011). Approximately 80 per cent of the general population exhibits optimism bias, which persists even when confronted with disconfirming evidence. Empirical research has demonstrated that compared to non-smokers and ex-smokers, smokers minimise the mortality consequences of smoking and the addictive nature of nicotine (Masiero et al. 2015; Leventhal et al. 1987).

Anchoring fallacy, as described by Tversky and Kahneman (1974), refers to the phenomenon in which individuals are heavily influenced by the first piece of information they encounter (the anchor). This bias occurs when an estimate is made by starting at an initial value and then adjusting it to yield a final answer, where the final answer is often biased towards the initial value. Goodin (1989) further explains that most smokers mistakenly conclude that smoking is safe for them, because they do not experience immediate perceptible harm from it.

The tendency to prioritise immediate rewards over future rewards is referred to as time discounting. When applied to smoking, a smoker values the immediate gratification derived from smoking a cigarette over the long-term benefits of abstaining, despite the fact that the value of long-term benefits is considerably greater. Neil Levy has proposed a comprehensive account of time discounting, suggesting that unwanted addition is characterised by the oscillation of preferences of the addict. Typically, an unwilling addict disavows their addiction repeatedly and wishes to abstain; however, they regularly change their minds and consume instead (Levy 2006). Levy employs the theory of hyperbolic discounting, a behavioural economics concept, to demonstrate how an addict, when presented with the opportunity of consumption or when experiencing cravings, discounts the future benefit of abstentinence in favour of the imminent pleasure of consumption (Levy 2006; Monteresso and Ainslie 2007).

**Fig. 1:**
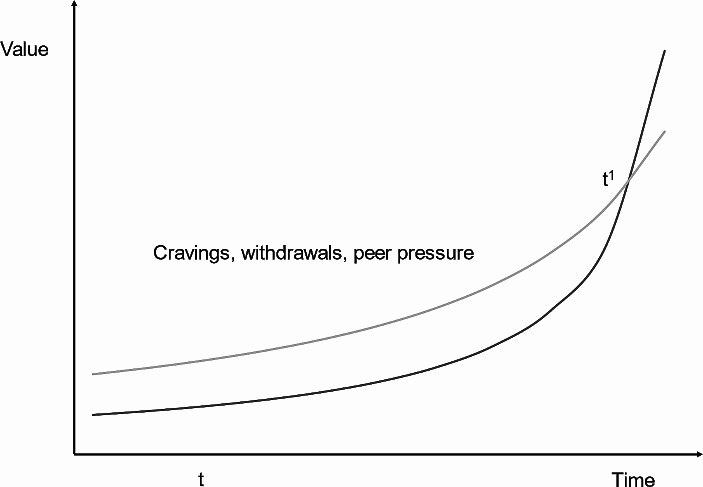
Hyperbolic curves drawn according to the formula Present value = Value_0_ / [1 + (k x Delay)] where Value_0_ is the value if the reward was immediate and *k* is the degree of impatience. The light grey curve represents future health and the dark grey curve represents smoking. *Adapted from* Ainslie (2010)

Levy posits that hyperbolic curves can cause discount curves to intersect. If this concept is applied to smokers, it can be illustrated as in Fig. 1: there are two curves, one representing health (curve X, light grey) and the other representing smoking (curve Y, dark grey). A smoker recognises the benefits of smoking and abstention, motivating them to prioritise health over smoking at a specific time, t. However, as time progresses, nicotine addiction triggers cravings, cues and withdrawal symptoms. Hyperbolic discounting explains how as time passes and smoking becomes more compelling and accessible, curve Y becomes steep and approaches curve X. At the decision point t^1^, the curves intersect, the value of smoking surpasses the value of health, and the smoker chooses to smoke. They then regret their decision and return to their original weighting of the two curves only to repeat the cycle once again. According to Levy, this explanation of addiction is superior to that of Frankfurt, as he elucidates how an addict can genuinely prefer consumption to abstinence while simultaneously wanting to abstain (2006).

Goodin (1989) argues that these cognitive deficits constitute a weak form of irrationality. Sarah Conly’s (2012) approach to cognitive bias calls for “coercive paternalism” as a means of addressing public health concerns like tobacco and obesity. She argues that smoking is a situation in which cognitive biases reveal the fallibility of humans in making decisions that may not be in our best interest.

The analysis of health-related behaviour through the lens of cognitive bias provides a useful framework for understanding why rational individuals engage in self-destructive behaviours. By characterising smoking in this manner, we humanise individuals without necessarily judging them as being uninformed, undisciplined, or lacking in knowledge.

### Psychosocial theories in addiction

Psychosocial theory explains self-understanding, identity formation, social relationships, and one’s worldview as a product of interactions among biological, psychological, and societal systems (Newman and Newman 2020). Development is the result of continuous interactions between individuals and their social environments. At each life stage, a normative crisis arises as an individual struggles with the gap between their existing competencies and the new demands of society. Resolving this tension involves the use of familiar coping strategies while learning new ones. A positive resolution enhances an individual’s capacity to adapt successfully to the succeeding stages. A negative resolution impairs an individual’s ability to adapt and thrive in the future.

The idea of core pathologies is one aspect of psychosocial theory, which posits that these destructive forces emerge from ineffective and negative resolution of crises at each developmental stage. Examples of such pathologies include poor attachment in infancy, which can manifest as social and emotional detachment and infant withdrawal; in toddlerhood, a lack of control over expressions of autonomy can lead to compulsion and repetitive behaviors driven by impulse or the restriction of impulses; and in older children, frustration over taking initiative can result in inhibition and psychological restraint that impedes freedom of thought, expression, and activity (Newman and Newman 2020).

Gabor Maté, in his book “In the Realm of Hungry Ghosts”, brings together neuroscience, psychosocial theory and his clinical experience as a family physician working in drug addiction to explore the underlying causes of addiction (2008). According to Maté, addicts’ brains exhibit less white and grey matter, which impairs their ability to acquire new information, adapt to circumstances, and engage in rational thought. Specifically, drug use affects the dopamine-motivation system, endogenous opioid circuity, and orbitofrontal cortex – brain regions that are crucial for decision-making, impulse control, and emotional regulation. Consequently, an impaired brain leads to deficits in an individual’s cognitive processes, behaviour, and emotional life. Maté emphasises the necessity for the brain, the organ responsible for decision-making, to initiate its own recovery. He asserts that “an altered and dysfunctional brain must decide that it wants to overcome its own dysfunction” (Maté 2008).

A key feature of Maté’s theory is that addicts’ brains are predisposed to dysfunction, even before their first drug use experience (2008). According to him, addiction stems from impaired brain development during fetal development, infancy, and early childhood. The environment in which a child grows shapes their brain development. The proper maturation of dopamine, opioid and orbitofrontal cortex circuits is contingent on a healthy environment that includes physical security and consistent emotional nurturing. Positive interactions between children and caregivers promote endorphin release, which fosters attachment relationships and the development of brain circuitry. The strength of attachment relationships directly affects the health of these circuits. The ability of a child to cope with psychological and physiological stress is largely dependent on their relationship with their caregiver. This ability is gradually acquired in life, with infants being entirely reliant on their caregivers to overcome stress. Maldevelopment occurs when infants and young children do not experience consistently secure interactions or are exposed to an excessive number of stressful ones. Compared to children who have good attachment relationships and are well nurtured, children with poor attachment relationships do not have the same opportunities to learn this life skill.

In conclusion, addiction-susceptible individuals typically exhibit immature decision-making, impaired rational thinking, and difficulty in emotional and stress regulation. These factors contribute to an increased likelihood of developing addiction. The addict’s brain is essentially damaged, rendering them more inclined to seek relief from stress from external sources, including from drugs that provide mood-enhancing effects. This explains two interconnected phenomena. Firstly, individuals who experience trauma exhibit a higher prevalence (Mills et al. 2006) and risk of addiction (Felitti et al. 1998), including addiction to nicotine (Budenz et al. 2021; Yoon et al. 2020). Secondly, social factors, such as racism, immigration, and housing instability, increase the risk of addiction (Amaro et al. 2021), thereby accounting for disproportionate prevalence and impact of addiction on socially disadvantaged populations (Greenhalgh et al. 2021; Pear et al. 2019).

In the context of this discussion, making free choices can be seen as problematic. According to Maté (2008), freedom entails choosing long-term spiritual and physical well-being over short-term pleasures. However, rational thought is often overpowered by unconscious forces and automatic brain circuitry. When comparing addiction to obsessive-compulsive disorder, Maté posits that the choices made in addiction are driven by unconscious emotional impulses or subliminal beliefs, which are programmed during early childhood. The stronger the unconscious part of the brain and the weaker the parts that control conscious thought, the less capacity an individual has for free and rational decision-making.

According to Van der Eijk and Uusitalo (2016), addiction is best understood through a psychosocial approach that emphasises a sociorelational understanding of autonomy. It is not solely the result of an individual’s preferences and motivations or solely the consequence of the biological effects of repeated drug use. Understanding the psychosocial factors at play is critical to comprehending the exercise of choice and autonomy in any moral agent, particularly in one struggling with addiction. Their conceptual framework divides environments into autonomy-promoting environments, such as those with emotional support, meaningful life opportunities, and low levels of stress, and autonomy-undermining environments, characterised by high levels of emotional pain, minimal opportunities, and high levels of stress. By providing individuals with the right environment, addicts and addiction-susceptible individuals become better equipped to make free and informed choices. Understanding psychosocial theories has led to shifts in the understanding of, and approaches to, drug addiction. The decriminalisation of some drug use and the use of harm reduction strategies have, in part, come from a more holistic understanding of addiction.

This approach places significant importance on childhood trauma and is drawn from observational studies and not large-scale empirical evidence. Questions also arise regarding the generalisability of the theory to nicotine, as a considerable portion of the evidence is based on more severe drug use in the form of cocaine, heroin and alcohol. According to Maté, chronic injecting drug use represents the most extreme end of the continuum, and although lower levels of disruption in childhood experiences and brain development may occur, they still result in milder forms of addiction or non-drug behavioral addictions (2008).

## Smoking, a nonautonomous act

In the previous sections, I have illustrated several points. First, a fundamental prerequisite for autonomy is that there exists a certain level of liberty, self-reflection, and agency. Second, nicotine addiction is a complex condition. It involves both nicotine’s direct effect on brain functioning and conditioned behaviour, as well as its exacerbation of cognitive bias, which reinforces addiction. Lastly, understanding addiction from a psychosocial and trauma-informed perspective highlights impaired brain development, emotional drivers, and subconscious thoughts as crucial factors in determining the choices and actions of individuals.

Smoking is not an autonomous act. The effect of nicotine on brain circuits and its involvement in conditioning behaviour related to nicotine circumvents self-reflection of volitions in favour of strong internal biological preferences. Even when an individual rationally decides to resist cravings and manage withdrawal symptoms, oscillating preferences cause smokers to weigh the value of immediate pleasure against the value of long-term health. Understanding human brain development and the impact of trauma on the risk of addiction is crucial for comprehending the decision-making difficulties experienced by addicts and addiction-susceptible individuals. Physical and psychological compulsions to smoke block the normative competency required to evaluate decisions.

Overcoming addiction is a complex undertaking that is more than an exercise of better rational thinking or a greater exercise of will power. Psychosocial theories and the effect of trauma on the developing brain necessitate a more critical analysis of free will and decision-making.

Reasoning through a decision involves a careful and logical appraisal of the benefits and risks associated with a choice. It also involves acknowledgement of factors that may influence choice, such as peer pressure, emotions, and cognitive bias. Despite being aware of the harmful effects of smoking, smokers act irrationally by continuing their habit. They may be aware of the impact of nicotine on the brain, but they are unaware of the influence of their social environment and the deeper psychological and emotional factors that drive their behavior. Consequently, they struggle to think and act independently, and their ability to engage in rational thought is impaired, hindering their decision-making.

Respecting the autonomy of smokers when it comes to smoking is tenuous. If we accept that smokers lack autonomy in their choice to smoke, using autonomy as grounds to oppose a smoking ban is weak. However, this stance is not as controversial as it might seem. While Mill is well known for defending the right to behave as one pleases as long as it does not harm others, he was not opposed to paternalism per se (Brink 2009). Mill believed that a person choosing slavery cannot be considered autonomous, as this would be contrary to promoting freedom. Archard (1990) explains that Mill valued the exercise of individual freedom which is self-abrogated by voluntary slavery. A society cannot intrinsically value freedom and autonomy, while simultaneously allowing for its self-repudiation. As Mill (2017) wrote, the principle of freedom cannot require that he should be free to not be free”.

Contemporary liberals argue similarly. Raz (2003) posits that protection of autonomy necessitates the recognition of other duties. Raz’s explication of the harm principle is broader than Mill’s as it explicitly includes harm to one’s self, where harm is defined as that which impaires an individual capcity to pursue a good life and cultivate autonomy.

The types of actions that justify restrictions on freedom should be examined. This approach was adopted by Leclerc and Herrera (1999) when assessing the ethics of boxing. Chronic traumatic encephalopathy, a disease resulting from repetitive traumatic injuries to the brain, causes decline in cognitive function, depression, poor impulse control, and dementia (Stern et al. 2011). This raises the question of how boxing affects a boxer’s ability to make decisions, including their decision to engage in and continue boxing. Leclerc and Herrera argue that evidence indicates that boxing places individuals at risk of making choices that impede their ability to flourish in life. As a result, they morally condemn the sport.

I make similar claims with regards to smoking. The evidence demonstrates that smoking impairs an individual’s ability to act autonomously when smoking. Nicotine addiction prevents moral agents from acting freely, and it is reasonable to restrict actions that infringe upon our liberty. Therefore, placing severe limitations on the choice to smoke is justified.

It does not immediately follow that accepting smoking as a nonautonomous act necessitates a complete ban on smoking. Such an action would harm nicotine addicts and is unrealistic and unsustainable. To determine the kind of approach that should be used to address a public health need, Conly (2012) provides four conditions required for implementing paternalism in healthcare. These are:

1. The activity being prevented is opposed to our long-term ends.
2. The coercive measure must prove effective in achieving its intended purpose.
3. The benefits of the measure outweigh the costs.
4. The measure must be the most effective way to prevent the activity in question.

A complete smoking ban is likely to contravene conditions 2 and 4. However, the legislations proposed in New Zealand and Malaysia were not absolute prohibitions on smoking, but rather generational smoking bans, that targeted potential future smokers. A generational ban is likely to be the most effective means of reducing the prevalence of smoking, as it addresses a non-addicted population (Department of Health & Social Care 2023; Ouakrim 2023). This approach also overcomes the limitations of current measures, such as tax increases and public health education campaigns, which face challenges and are close to their limits of effectiveness (Berrick 2013; Khoo et al. 2010). Public health experts are training their focus on the group at the highest risk of becoming smokers, adolescents. For instance, Malaysia is experiencing an increase in adolescent smokers (Mohd Yusoff et al. 2022) and the mean age of smoking onset is decreasing (Lim et al. 2013).

We must acknowledge that philosophically and ethically speaking, a comprehensive smoking ban is consistent with upholding autonomy. The reasons underpinning this position also apply to individuals who have not started smoking, so I extend my argument to generational smoking bans. By preventing individuals from becoming smokers, we can preempt the conditions that impair rational thought and thus undermine autonomy. Given the impracticalities of a complete smoking ban, a generational ban will be more effective, leading to a greater reduction in smoking prevalence and related public health outcomes. The novelty of my argument lies in the use of evidence from neurobiology, psychology, and psychosocial development to demonstrate impaired decision-making that renders smoking nonautonomous. It is crucial to emphasize that this stance is grounded in promoting and protecting autonomy rather than sacrificing it for competing considerations.

## Criticisms

I have identified two aspects of my argument that could be challenged by those who argue against a generational smoking ban on the basis of free choice and autonomy.

The first criticism of my argument is that irrationality is incompatible with autonomy. This perspective is based on the premise that rationality and autonomy are two separate concepts, and that respecting autonomy calls necessitates embracing irrational decisions. Indeed, in ideal forms of autonomy, such as in Kant’s philosophy. Indeed, in ideal forms of autonomy like with Kant, “pure reason” is a requirement of autonomy (O’Neill 2002). Although rationality is not explicitly mentioned as a criterion in contemporary notions of autonomy, it is implicitly implied in terms like “agency,” “intentionality” and “self-reflection.” Several bioethicists, including Savulescu (1994) and O’ Neill (2002), also emphasize the importance of rationality in the exercise of autonomy.

Nevertheless, it is necessary to reconcile allowing individuals to make irrational choices without always labelling them as lacking autonomy. When we allow individuals to make irrational choices and act on them, we do not believe it is morally right to make poor decisions; rather, we aim to enhance their capacity for autonomous action in the future. Respecting the decisions of individuals at present fosters a therapeutic relationship and facilitates experiential learning, both of which may alter perspectives and promote rational decision-making in the future. In many instances of irrational decision-making, preventing the consequences of such decisions is impractical, such as coercing a patient to undergo medical treatment. Moreover, the majority of irrational decisions that we allow individuals to make result in harms that are either minimal or self-inflicted. It is only when these harms are significant and widespread that the threshold for intervention is reduced, as in public health emergencies. When we speak of respect for autonomy, we refer to respect for individuals as beings capable of autonomy, not respect for individual decisions.

Walker (2009) presents a theory of rational autonomy in which she differentiates between autonomous beings and autonomous decisions. According to her, adults who are otherwise competent and respected as autonomous beings can nevertheless fail in reasoning and make nonautonomous decisions. However, this does not always warrant paternalistic intervention, as she separates the moral obligation to abide by decisions made by autonomous individuals from the moral obligation to respect their autonomy.

I contend that autonomous individuals make nonautonomous decisions pertaining to smoking. This does not mean that we do not respect them as autonomous beings nor does it mean that we force them into abstinence. We respect the autonomy of smokers as autonomous individuals, just not when it comes to the decision to smoke. For those who have not started smoking, measures can be taken to severely restrict access to cigarettes.

The second objection to my argument is that health is not a universal value. Proponents of libertarianism contend that individuals should bear the responsibility for their own health choices with minimal government interference and that the free market should devise solutions. Nevertheless, health outcomes in countries such as the United States tend to rank poorly among developed nations. This criticism overlooks the fact that health and public health are shared goals.

Health is valued as an end in itself, or as a means to an end. Achieving good health enables individuals to live longer and in better conditions, which, in turn, allows them to pursue other ends such as success and financial independence. Public health is essential for several reasons, including the need for large-scale interventions that require significant resources, financial investment, and collective action, which are beyond the capacity of individuals acting alone. As the community, we have developed concepts such as universal healthcare, made healthcare a human right, and created systems in which to protect the health of the vulnerable. While there may be debates regarding the specifics of what good health entails, it is difficult to conceive of individuals and a society in which good health is not pursued.

A majority of smokers aspire to stop smoking (Babb et al. 2017), and a considerable number attempt to do so (Australian Institute of Health and Welfare 2020). Avoiding smoking-related harms leads to good health. The significance of good health and public health as universal values is evidenced by the considerable weight that governments, institutions and individuals afford them. This provides compelling grounds for asserting that they are foundational societal values. Reducing smoking-related harms is a powerful tool to achieve this objective.

## Conclusion

Reducing smoking-related harms is a public health priority and generational smoking bans are a means to achieve this goal. I have defended a generational smoking ban from one of its strong ethical and political objections, that the policy undermines autonomy. I have done so by showing that the basic conditions of autonomy are not met by smokers because of the inability of the brain to make rational and free decisions due to nicotine addiction, cognitive bias and the effects of trauma on psychosocial development.

I uphold that autonomy is a principle of a liberal state that requires protection. A generational smoking ban is compatible with a concern for autonomy on ethical and philosophical grounds. Advocates of liberalism who consider tobacco a public health priority and are concerned about the welfare of future generations should support a generational smoking ban, confident in the knowledge that they are not betraying fundamental liberal principles. Undoubtedly, this policy is a bold move, but it is entirely consistent with the founding principles of liberalism.
